# Genome-wide association study using deregressed breeding values for cryptorchidism and scrotal/inguinal hernia in two pig lines

**DOI:** 10.1186/s12711-015-0096-6

**Published:** 2015-03-21

**Authors:** Claudia A Sevillano, Marcos S Lopes, Barbara Harlizius, Egiel HAT Hanenberg, Egbert F Knol, John WM Bastiaansen

**Affiliations:** Topigs Norsvin, PO Box 43, 6640 AA Beuningen, the Netherlands; Animal Breeding and Genomics Centre, Wageningen University, PO Box 338, 6700 AH Wageningen, the Netherlands

## Abstract

**Background:**

Cryptorchidism and scrotal/inguinal hernia are the most frequent congenital defects in pigs. Identification of genomic regions that control these congenital defects is of great interest to breeding programs, both from an animal welfare point of view as well as for economic reasons. The aim of this genome-wide association study (GWAS) was to identify single nucleotide polymorphisms (SNPs) that are strongly associated with these congenital defects. Genotypes were available for 2570 Large White (LW) and 2272 Landrace (LR) pigs. Breeding values were estimated based on 1 359 765 purebred and crossbred male offspring, using a binary trait animal model. Estimated breeding values were deregressed (DEBV) and taken as the response variable in the GWAS.

**Results:**

Heritability estimates were equal to 0.26 ± 0.02 for cryptorchidism and to 0.31 ± 0.01 for scrotal/inguinal hernia. Seven and 31 distinct QTL regions were associated with cryptorchidism in the LW and LR datasets, respectively. The top SNP per region explained between 0.96% and 1.10% and between 0.48% and 2.77% of the total variance of cryptorchidism incidence in the LW and LR populations, respectively. Five distinct QTL regions associated with scrotal/inguinal hernia were detected in both LW and LR datasets. The top SNP per region explained between 1.22% and 1.60% and between 1.15% and 1.46% of the total variance of scrotal/inguinal hernia incidence in the LW and LR populations, respectively. For each trait, we identified one overlapping region between the LW and LR datasets, i.e. a region on SSC8 (*Sus scrofa* chromosome) between 65 and 73 Mb for cryptorchidism and a region on SSC13 between 34 and 37 Mb for scrotal/inguinal hernia.

**Conclusions:**

The use of DEBV in combination with a binary trait model was a powerful approach to detect regions associated with difficult traits such as cryptorchidism and scrotal/inguinal hernia that have a low incidence and for which affected animals are generally not available for genotyping. Several novel QTL regions were detected for cryptorchidism and scrotal/inguinal hernia, and for several previously known QTL regions, the confidence interval was narrowed down.

**Electronic supplementary material:**

The online version of this article (doi:10.1186/s12711-015-0096-6) contains supplementary material, which is available to authorized users.

## Background

Cryptorchidism and scrotal/inguinal hernias are the most frequent congenital defects in pigs with incidences that range from 0.27 to 0.90% and 0.39 to 1.09% for cryptorchidism and scrotal/inguinal hernia, respectively [1-3]. Cryptorchidism occurs when the testis fails to descend from its intra-abdominal location into the scrotum [4]. An inguinal hernia occurs when part of the small intestine passes through the internal inguinal ring and is present in the inguinal canal. A scrotal hernia occurs when part of the small intestine passes all the way through the inguinal canal and enters the scrotum [5,6]. Distinguishing between scrotal and inguinal hernias is not easy without clinical examination [7], therefore these two traits were considered as a single trait, hereafter called ‘hernia’.

Identification of genomic regions that control these congenital defects is of great interest to breeding programs, both from an animal welfare point of view and for economic reasons. Many studies have aimed at identifying regions in the genome that can, partially, explain the occurrence of these defects [5,8,9] but success was limited because of insufficient power in the analyses due to the small number of samples and/or the study design (e.g. parent-offspring trios, affected sib pairs).

Here, a novel approach was implemented to perform a genome-wide association study (GWAS) for congenital defects. To obtain “phenotypes” of the genotyped animals, we applied a pedigree-based method to calculate estimated breeding values (EBV). These EBV were subsequently deregressed (DEBV) and used as response variable in the GWAS analysis. The use of DEBV obtained with a binary trait model was considered to be a powerful approach for traits like cryptorchidism and hernia, which have a low incidence and for which affected animals are generally not genotyped [10]. Our aim was to identify single nucleotide polymorphisms (SNPs) associated with cryptorchidism and hernia using DEBV.

## Methods

### Ethics statement

The data used for this study was collected as part of routine data that are recorded in a commercial breeding program. Samples collected for DNA extraction were only used for routine diagnostic purposes of the breeding program. Data recording and sample collection were conducted strictly in line with the Dutch law on the protection of animals (Gezondheids- en welzijnswet voor dieren).

### Phenotypes

The phenotypes consisted of 1 360 453 records of purebred and crossbred male offspring of genotyped animals from two sow lines: Large White (LW) and Landrace (LR). Phenotypic data were collected from 57 different farms. Presence of cryptorchidism or hernia was recorded as either 1 (affected) or 0 (not-affected). In total, 2947 pigs were recorded with cryptorchidism and were offspring of 832 sires and 2287 dams with one to 34 affected offspring per sire and one to six affected offspring per dam. In total, 5251 pigs were recorded with hernia and were offspring of 995 sires and 3859 dams with one to 115 affected offspring per sire and one to eight affected offspring per dam. Of the 184 239 litters, 2599 were affected by cryptorchidism and 4446 were affected by hernia with one to six affected pigs per litter for both defects. None of the pigs were affected by both cryptorchidism and hernia.

### Genotypes

For the GWAS, animals that were genetically related with the phenotyped animals were genotyped. Thus, 2570 LW pigs (608 males and 1962 females) and 2272 LR pigs (835 males and 1437 females) were genotyped using the Porcine SNP60 Beadchip of Illumina (San Diego, CA, USA) [11]. Of the 2570 genotyped LW pigs, 2264 had records from their own offspring. Genotyped sires had on average 1093 individual offspring records that ranged from one to 6128 per sire, while genotyped dams have on average 38 individual offspring records that ranged from one to 150 per dam. Of the 2272 genotyped LR pigs, 1825 had records from their own offspring. Genotyped sires had on average 903 individual offspring records that ranged from four to 5438 per sire, while genotyped dams had on average 32 individual offspring records that ranged from one to 119 per dam.

Genotype data were filtered in a two-step quality control process. First, SNPs were removed if their GenCall score was less than 0.15, if they were located on SSCY (SSC for *Sus scrofa* chromosome), or if their position on the genome build10.2 was unknown [12]; animals were removed if they had a missing genotype frequency of 0.30 or higher. Then, SNPs with a call rate less than 0.95, a minor allele frequency less than 0.01 and/or a strong deviation from Hardy Weinberg equilibrium (*χ*^2^ > 600) were removed. For males, SNPs that were outside the pseudoautosomal region on SSCX were also removed if the frequency of heterozygous calls was greater than 0.05. Animals with a missing genotype frequency higher than 0.05 were removed.

Genotyped parent-offspring pairs were checked for Mendelian inconsistencies. Offspring and parents with more than 1% Mendelian inconsistencies with each of their parents or offspring were excluded.

After quality control, genotyping data of the LW and LR populations consisted of 2102 and 1926 animals, and 38 632 and 39 508 SNPs, respectively. The final call rate was 99.6% for both populations.

### Breeding value estimation

For all phenotyped animals, pedigree information was collected up to 20 generations back. The pedigree data included 1 434 713 animals. A binary single trait animal model was used to estimate heritabilities and EBV by restricted maximum likelihood methodology implemented in the software ASReml [13]. The following model was applied:

1 $$ {Y}_{ijklmn}=\mu +HY{S}_i+TN{B}_j+{P}_k+L{L}_l+{c}_m^2+{a}_n+{e}_{ijklmn}, $$

where *Y*_*ijkmn*_ was the phenotype for cryptorchidism or hernia observed in animal n, *μ* was the overall mean, *HYS*_*i*_ was the fixed effect of herd-year-season of birth *i* (1194 classes), *TNB*_*j*_ was the fixed effect of number of littermates *j* (30 classes), *P*_*k*_ was the fixed effect of the parity *k* of the mother (seven classes) and *LL*_*l*_ was the fixed effect of litter type *l* (four classes i.e. LWxLW, LWxLR, LRxLR, LRxLW). Random effects included the common litter effect (${c}_m^2$) assumed to be normally distributed ~ *N*(0, **I**${\sigma}_{\mathrm{c}}^2$), where **I** is an identity matrix and ${\sigma}_{\mathrm{c}}^2$ is the unknown variance between litters. The additive genetic effect (a_n_) assumed to be normally distributed ~ *N*(0, **A**${\sigma}_{\mathrm{a}}^2$), where **A** was a known matrix of additive genetic relationship among animals and ${\sigma}_{\mathrm{a}}^2$ was the unknown genetic variance between animals. The residual error (*e*_*ijklmn*_) was defined on the logistic scale by setting the residual variance to 1.

The EBV obtained with model 1 were deregressed according to Garrick et al. [14], whereby parental average effects were removed as part of the deregression process to obtain a more accurate estimate of the expected phenotype. In addition, following the approach of Garrick et al. [14], a weighting factor (*w*) was estimated based on the reliability of the calculated DEBV by considering a value of 0.5 for the scalar c.

### Association analysis

A single SNP GWAS was performed with the software ASReml [13] by applying the following model:

2 $$ DEB{V}_{ij}\ w=\mu +SN{P}_i+{a}_j+{e}_{ij}, $$

where *DEBV*_*ij*_ is the DEBV for genotyped animal *j*, *μ* is the overall DEBV mean of the genotyped animals, *SNP*_*i*_ is the genotype of the SNP *i* coded as 0, 1 or 2 copies of one of the alleles, *a*_*j*_ is the additive genetic effect and *e*_*ij*_ the residual error. The weighting factor *w* was used in the GWAS to account for differences in the amount of available information on offspring to estimate DEBV [14].

Only animals with a *w* value higher than 0 and a minimum reliability of DEBV of 0.06 for cryptorchidism and 0.08 for hernia were included in the GWAS. DEBV of 1528 LW and 1229 LR pigs for cryptorchidism and DEBV of 1361 LW and 1405 LR pigs for hernia were included in the GWAS.

To account for multiple testing, a false discovery rate (FDR) implemented in the R package ‘qvalue’ [15] was applied. A FDR ≤ 0.20 was set to define significant associations.

### QTL regions

QTL regions were defined based on the location of the significant SNPs. All significant SNPs located within a region of 10 Mb were considered to belong to the same QTL region. For multiple QTL regions to be considered present on the same chromosome, the distance between consecutive significant SNPs had to be larger than 10 Mb. The genetic variance explained by each QTL region (${\sigma}_{SNP}^2$) was calculated as follows:

$$ {\sigma}_{SNP}^2 = 2pq\times {\alpha}^2, $$

where *p* is the observed allele frequency of the top SNP in each QTL region, *q* = 1-*p* and *α* is the estimated allele substitution effect obtained from model 2. The result was expressed as the percentage of the total variance explained by the $\mathrm{S}\mathrm{N}\mathrm{P}\left(\frac{\upsigma_{\mathrm{SNP}}^2}{\upsigma_{\mathrm{P}}^2} \times 100\right)$.

### Candidate genes

Putative candidate genes within the QTL regions and in the neighbouring upstream and downstream 2-Mb regions were identified based on the Sscrofa10.2 genome assembly, using the NCBI Map Viewer (http://www.ncbi.nlm.nih.gov/projects/mapview/).

## Results and discussion

For GWAS to be successful, it is essential to obtain a relevant dataset, which in the case of congenital defects is especially challenging because of their low incidence, and because affected animals are generally culled when diagnosed and thus not included in sampling and genotyping. For this study, we combined phenotypes from many thousands of animals with genotypes of their ancestors. To connect genotype to phenotype data, phenotypic observations of individuals were used to estimate the EBV of their genotyped ancestors.

### Breeding value estimation and variance components

Incidences of cryptorchidism and hernia were on average 0.22% and 0.39%, respectively. The incidence of cryptorchidism was lower in LW than in LR pigs (0.14 vs. 0.33%), while the incidence of hernia was slightly higher in LW than in LR pigs (0.42% vs. 0.34%) (Table 1). Concerning litter type, the highest incidence of cryptorchidism and hernia was observed in purebred litters of both lines compared to their corresponding crossbred litters (Table 1).

**Table 1:** **Mean incidence (%) of cryptorchidism and hernia per sow line and litter type**

**Sow line**	**Litter type**	**Hernia (%)**	**Cryptorchidism (%)**
Large White	Purebred	0.974	0.426
	Crossbred	0.260	0.066
	Combined	0.415	0.144
Landrace	Purebred	0.352	0.551
	Crossbred	0.338	0.252
	Combined	0.342	0.331
Overall		0.386	0.217

Heritabilities for cryptorchidism and for hernia were estimated at 0.26 ± 0.02 and 0.31 ± 0.01, respectively, using an animal model (Table 2). In the literature, heritability estimates reported for congenital defects range from 0.03 to 0.27 [1-3,9,16] depending on the population sampled and the statistical model (usually obtained with a sire-model [1-3,9] except for [16]). Estimation of the heritability of binary traits is not straightforward and contradictory results have been reported, although the factors responsible for these differences remain unclear [17]. Moreover, Moreno et al. [18] showed that, in models for binary traits, inferences about the variance component of random effects are biased when there is little information on fixed effects, which leads to poor estimates of variance components. The mean of the posterior distribution of the variance component when using the animal model, is biased upwards and leads to overestimation of the heritability. With our large dataset, we obtained more precise estimates of fixed effects (herd-year-season, number of littermates, litter line, and parity), thus reducing the potential for biased estimates of variance components.

**Table 2:** **Additive genetic variance (** ${\boldsymbol{\upsigma}}_{\mathbf{A}}^2$ **), common litter effect (c** ^2^ **) and heritability (h** ^2^ **) for cryptorchidism and hernia**

**Trait**	**σ** ^2^_A_	**c** ^2^	**h** ^2^
Hernia	1.69 (0.09)	0.53 (0.06)	0.31 (0.01)
Cryptorchidism	1.41 (0.11)	0.64 (0.08)	0.26 (0.02)

Standard errors are in parenthesis; effects are under an underlying logistic scale.

The common litter environment (c^2^) was found to explain an important part of the variance for cryptorchidism and hernia. The proportion of variance due to c^2^ was estimated at 0.64 for cryptorchidism and 0.53 for hernia. Thaller et al. [3] reported a significant c^2^ effect that improved remarkably the fit of a binary model to data on congenital defects in pigs. They also suggested that intrauterine and possibly postnatal environments substantially influence the occurrence of these disorders. If the sow is exposed to disease during gestation, and subsequently to medical treatment, or if it is in contact with toxic products such as mycotoxins in the feed, the development of the piglets can be affected, and an increase in the incidence of congenital defects in those litters can be observed.

### QTL regions and candidate genes

#### *Cryptorchidism*

**Figure 1:**
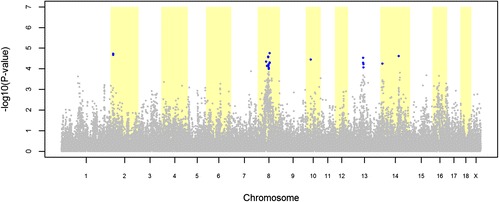
**Association between cryptorchidism and 38 632 genome-wide SNPs in a Large White pig population.** Each dot represents one SNP; on the y-axis are -log10(P-values) and on the x-axis are the physical positions of the SNPs by chromosome; blue dots represents SNPs that surpassed the FDR ≤ 0.20 threshold.

**Figure 2:**
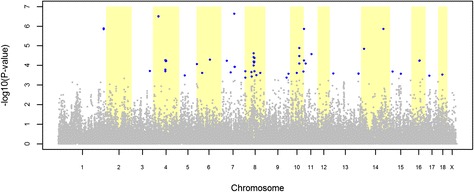
**Association between cryptorchidism and 39 508 genome-wide SNPs in a Landrace pig population.** Each dot represents one SNP; on the y-axis are -log10(P-values), and on the x-axis are the physical positions of the SNPs by chromosome; blue dots represents SNPs that surpassed the FDR ≤ 0.20 threshold.

The GWAS for cryptorchidism identified 22 and 83 significant SNPs (FDR ≤ 0.20) in the LW and LR pig datasets, respectively (Figures 1 and 2). The quantile-quantile plots (QQ plot) for these GWAS are shown in Additional file 1: Figure S1. For the LW dataset, the 22 significant SNPs were distributed over seven QTL regions located on SSC2, 8, 10, 13 and 14 (Table 3). The most significant SNP of each QTL region explained between 0.96% and 1.10% of the total variance of cryptorchidism incidence in the LW population (Table 3). For the LR dataset, the 83 significant SNPs were distributed over 31 QTL regions located on all autosomes with the exception of SSC2 and SSC12 (Table 4). The most significant SNP of each QTL region explained between 0.48% and 2.77% of the total variance of cryptorchidism incidence in the LR population (Table 4).

**Table 3:** **QTL regions associated with cryptorchidism in the Large White population**

**Chromosome**	**Position (Mb)**	**SNP per QTL region**	**Proportion (%)**
SSC2	16.15 - 16.17	2	0.96
SSC8	43.93	1	1.06
SSC8	64.95 – 84.44	12	1.07
SC10	25.28	1	0.98
SSC13	77.69 - 79.80	4	1.09
SSC14	11.37	1	0.99
SSC14	96.99	1	1.10

Significant association using a FDR ≤ 0.20; proportion (%) of the phenotypic variance explained by the most significant SNP per QTL region.

**Table 4:** **QTL regions associated with cryptorchidism in the Landrace population**

**Chromosome**	**Position (Mb)**	**SNP per region**	**Proportion (%)**
SSC1	297.27 – 297.77	4	1.86
SSC3	124.83	1	2.08
SSC4	26.06 - 26.11	2	2.03
SSC4	75.99 – 79.65	15	1.01
SSC5	34.07	1	1.18
SSC6	86.23	2	1.25
SSC6	30.24	1	0.79
SSC6	3.66	1	2.34
SSC7	31.30	1	1.53
SSC7	51.03	1	1.41
SSC7	71.81 – 74.94	9	2.30
SSC8	3.61 – 3.82	2	1.49
SSC8	34.81- 36.22	2	1.70
SSC8	60.77 – 72.81	16	2.23
SSC8	87.00	1	1.39
SSC8	121.33	1	1.04
SSC9	137.13 – 144.94	2	1.74
SSC10	37.87	1	1.21
SSC10	54.50 – 55.68	3	2.77
SSC10	76.17	1	1.37
SSC11	2.68 – 13.27	3	1.24
SSC11	53.48	1	2.10
SSC13	17.67	1	1.46
SSC13	200.75 – 201.80	3	0.71
SSC14	15.42	1	2.04
SSC14	120.99	1	1.24
SSC15	15.08	1	1.59
SSC15	82.48	1	1.20
SSC16	47.25 – 49.84	2	0.48
SSC17	24.01	1	1.13
SSC18	28.17	1	1.09

Significant association using a FDR ≤ 0.20; proportion (%) of the phenotypic variance explained by the most significant SNP per QTL region.

One QTL region located on SSC8 between 61 and 73 Mb was found to overlap between the LW and LR datasets. This region contains the *gonadotropin-releasing hormone receptor* (*GNRHR*) and *alpha-fetoprotein* (*AFP*) genes. In mouse, a mutation in *GNRHR* was shown to initiate undescended testes in male carriers [19]. The AFP protein is known to interact with oestrogens and may modulate foetal responses to oestrogens. Thus, high foetal AFP levels may have a direct role in the occurrence of cryptorchidism; but also, indirectly, by inducing placental dysfunction [20]. Because this region on SSC8 was found to be associated with cryptorchidism in both LW and LR populations and because it contained strong candidate genes that have been shown to be involved in testes descent, it is likely that it has a role in the incidence of cryptorchidism in pigs.

On SSC13, a QTL region was identified for cryptorchidism in the LW dataset and a peak was also observed in this same region in the LR dataset, but it did not surpass the FDR threshold. This region contains a candidate gene i.e. the *GATA binding protein 2* (*GATA2*) that is located at 79 Mb and has been shown to play a critical role in urogenital development [21]. In addition, expression of the GATA2 protein is regulated by FOG2 (friend of GATA2), a transcriptional cofactor that is able to differentially regulate the expression of GATA-target genes and has been shown to be involved in normal testis development in a mouse model [22].

In the LW dataset, a second region on SSC8 around 43 Mb was identified that contains the *platelet-derived growth factor receptor-alpha like* (*PDGFRA*) and *tyrosine-protein kinase* (*v-KIT*) genes. Phosphorylation of *PDGFRA* and *v-KIT* have a role in the development and function of male gonads and are associated with cryptorchidism in humans [23,24] and with gonadal hypoplasia in cattle [25]. No evidence was found for additional candidate genes in the QTL regions identified for both datasets.

Previously, Stinckens et al. [9] reported an association study for cryptorchidism using the Porcine SNP60 Beadchip in different populations of LW and LR pigs. There is no overlap between our results and those of Stinckens et al. [10], who found associations on SSC12 and SSCX (A. Stinckens, personal communication). This may be explained by the different structure of their dataset that included affected parent-offspring trios and by the different populations used, although of the same breed.

#### *Hernia*

**Figure 3:**
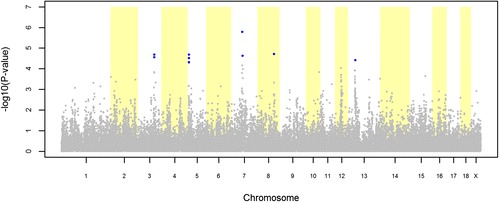
**Association between hernia and 38 632 genome-wide SNPs in a Large White pig population.** Each dot represents one SNP; on the y-axis are -log10(P-values), and on the x-axis are the physical positions of the SNPs by chromosome; blue dots represents SNPs that surpassed the FDR ≤ 0.20 threshold.

**Figure 4:**
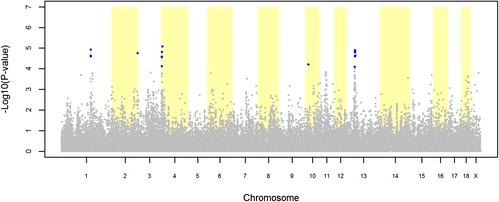
**Association between hernia and 39 508 genome-wide SNPs in a Landrace pig population.** Each dot represents one SNP; on the y-axis are -log10(P-values), and on the x-axis are the physical positions of the SNPs by chromosome; blue dots represents SNPs that surpassed the FDR ≤ 0.20 threshold.

The GWAS for hernia identified 10 and 22 significant SNPs in the LW and LR datasets, respectively (Figures 3 and 4). The QQ plots for these GWAS are shown in Additional file 1: Figure S1. For the LW dataset, the 10 significant SNPs were distributed over five QTL regions on SSC3, 5, 7, 8 and 13 (Table 5). The most significant SNP of each QTL region explained between 1.22% and 1.60% of the total variance of hernia incidence in the LW population (Table 5). For the LR dataset, the 22 significant SNPs were distributed over five QTL regions on SSC1, 2, 4, 10, and 13 (Table 6). The most significant SNP of each QTL region explained between 1.15% and 1.46% of the total variance of hernia incidence in the LR population (Table 6).

**Table 5:** **QTL regions associated with hernia in the Large White population**

**Chromosome**	**Position (Mb)**	**SNP per region**	**Proportion (%)**
SSC3	104.46 - 104.54	2	1.29
SSC5	5.04 – 5.36	4	1.22
SSC7	53.76 – 54.48	2	1.60
SSC8	119.72	1	1.31
SSC13	34.53	1	1.23

Significant association using a FDR ≤ 0.20; proportion (%) of the phenotypic variance explained by the most significant SNP per QTL region.

**Table 6:** **QTL regions associated with hernia in the Landrace population**

**Chromosome**	**Position (Mb)**	**SNP per region**	**Proportion (%)**
SSC1	162.16 – 162.80	3	1.41
SSC2	155.44	1	1.15
SSC4	53.96 – 71.57	5	1.46
SSC10	13.96	1	1.18
SSC13	33.62 – 37.15	12	1.26

Significant association using a FDR ≤ 0.20; proportion (%) of the phenotypic variance explained by the most significant SNP per QTL region.

One QTL region located on SSC13 between 34 and 37 Mb was found to overlap between the LW and LR datasets. This region was previously reported by Grindflek et al. [5] and Ding et al. [8]. It contains the *ras homolog family member A* (*RHOA*) gene, which regulates the contraction and shortening of smooth muscle tissues [26]. Smooth muscle cells play an important role in the obliteration of *processus vaginalis*, which is the embryological protrusion of the peritoneum that precedes testicular descent into the scrotum [27,28]. Incomplete obliteration of the *processus vaginalis* leads to cryptorchidism and hernia [28,29]. Altered expression of *RHOA* was identified in a rat strain with inherited cryptorchidism [30]. Since the region on SSC13 was shown to be associated with hernia in both LW and LR datasets studied here and in other previous studies [5,8], and because it contains a candidate gene that is connected with the process of obliteration of the *processus vaginalis*, it is likely to play a role in the incidence of hernia in pigs.

The region on SSC7 detected in the LW dataset overlaps with a region previously identified by Grindflek et al. [5]. In their paper, Grindflek et al. [5] suggested that *cytochrome P450 family 19A1* (*CYP19A1*) may be a candidate gene for this QTL, based on its location on the homoeologous chromosome in humans. However, according to the Ensembl Sscrofa10.2 database (http://www.ensembl.org/Sus_scrofa/Info/Index), the *CYP19A1* gene is located on SSC1 and not SSC7.

To our knowledge, it is the first time that the other three QTL regions identified on SSC3, 5, and 8 are found to be associated with hernia, although some studies [5,8] have reported that different regions on these chromosomes harbour QTL for hernia. The QTL region on SSC8 contains two candidate genes i.e., *pro-epidermal growth factor precursor* (*EGF*) and *lymphoid enhancer-binding factor 1* (*LEF1*). Mutations in *EGF* have been related to connective-tissue problems, such as inguinal hernia [31]. Regarding the putative role of the *LEF1* gene in hernia, it has been shown that the LEF1 protein constitutively associates with β-catenin which mediates the action of the anti-mullerian hormone (AMH) [32], itself involved in the gubernaculum swelling reaction that occurs during the first phase of testicular descent [33,34]. No other candidate genes were identified.

In the LR dataset, the QTL region on SSC4 overlaps with a previously reported QTL by Ding et al. [8]. To our knowledge, it is the first time that the three other regions identified on SSC1, 2 and 10 are found to be associated with hernia, although previous studies [5,7,8] have reported different regions on these chromosomes as harbouring QTL for hernia but no candidate genes were detected.

Genetic differences between the populations analysed and differences in marker density and/or power of resolution between our study and those of Grindflek et al. [5] and Ding et al. [8] may explain the differences in the regions detected. The only previous analysis on hernia in pigs which used the Porcine SNP60 Beadchip is that of Stinckens et al. [9] but on other LW and LR populations. They reported a QTL for hernia on SSC5 without specifying its location and some associations on SSC6 and SSCX, which were not confirmed in our study (A. Stinckens, personal communication). As mentioned before, differences in the structure of their dataset and in the populations analysed may explain the absence of detection of overlapping regions.

In general, the proportion of total variance described by strongly associated SNPs for cryptorchidism and hernia is low. Moreover, the number of regions that were found to be significantly associated with cryptorchidism and hernia is large, which indicates that cryptorchidism is a disease affected by many genes with small effects as suggested by Stinckens et al. [9] and Thaller et al. [3]. Our results suggest a similar architecture for hernia. The polygenic character of these two traits makes it is difficult to identify the causative variations that are involved.

## Conclusions

The use of DEBV in combination with a binary trait model was found to be a powerful approach for the GWAS of difficult traits such as cryptorchidism and hernia that have a low incidence and for which affected animals are generally not available for genotyping.

We detected several novel QTL regions and the confidence interval of some previously known QTL regions was narrowed down. Overlapping QTL regions between the two pig datasets was limited to one QTL for each trait.

We identified several significant SNPs that can contribute to an index of SNPs for selection against cryptorchidism and hernia in pigs. However, given the small proportion of variance explained by these SNPs, it will be necessary to use genomic estimated breeding values to expedite the reduction of cryptorchidism and hernia incidence in pig populations.

## Additional file

Additional file 1: Figure S1. Quantile-quantile plots for cryptorchidism for the Large White **(a)** and Landrace datasets **(b)** and for hernia for the Large White **(c)** and Landrace datasets **(d)**. Observed distribution of -log10(P-values) on the y-axis compared to the expected distribution of -log10(P-values) on the x-axis across the 38 632 and 39 508 SNPs for Large White and Landrace, respectively.
