# Supplementary material for: Genome-wide association study using deregressed breeding values for cryptorchidism and scrotal/inguinal hernia in two pig lines
Source: Genet Sel Evol. 2015 Mar 21;47(1):18. doi: 10.1186/s12711-015-0096-6 (PMC4367917; doi:10.1186/s12711-015-0096-6)
Supplement: Additional file 1: Figure S1. — Quantile-quantile plots for cryptorchidism for the Large White (a) and Landrace datasets (b) and for hernia for the Large White (c) and Landrace datasets (d). Observed distribution of -log10(P-values) on the y-axis compared to the expected distribution of -log10(P-values) on the x-axis across the 38 632 and 39 508 SNPs for Large White and Landrace, respectively. [file 12711_2015_96_MOESM1_ESM.pdf]

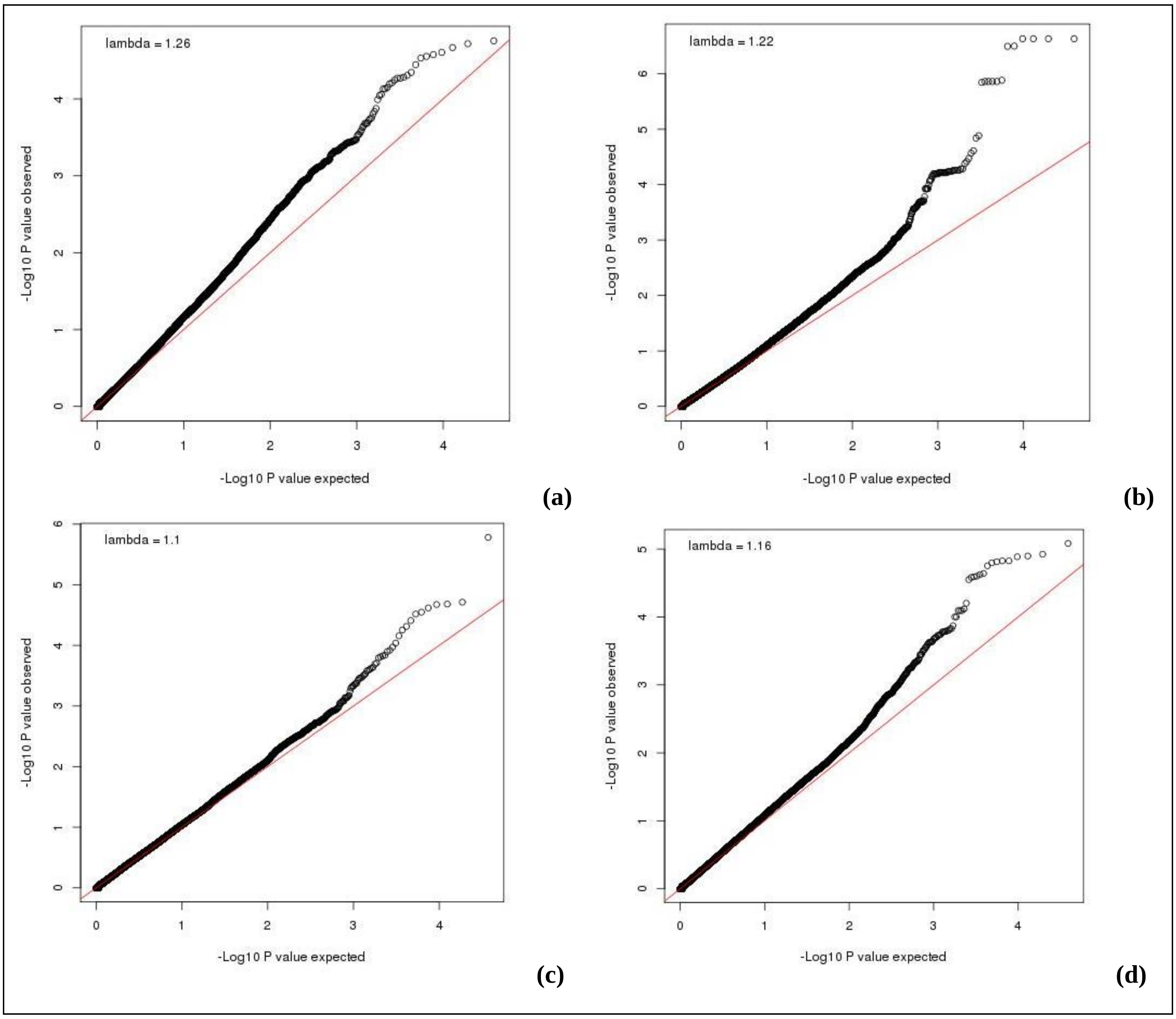

Quantile-quantile plots for cryptorchidism in Large White (a), for cryptorchidism in Landrace (b), for hernia in Large White (c), and for hernia in Landrace (d). Observed distribution of -log10(P-values) on the y-compare to the expected distribution of -log10(P-values) on the x-axis across the 38 632 and 39 508 SNPs for Large White and Landrace, respectively.
